# Supplementary material for: Study protocol for a randomized controlled trial comparing pulse pressure variation (PPV) and central venous pressure (CVP) guidance for fluid responsiveness assessment in neurosurgical patients undergoing posterior fossa tumor resection in park bench position
Source: PLoS One. 2025 Jun 2;20(6):e0324590. doi: 10.1371/journal.pone.0324590 (PMC12129314; doi:10.1371/journal.pone.0324590)
Supplement: S1 File — (DOCX) [file pone.0324590.s001.docx]

**Study protocol**

| Study Title | Fluid Responsiveness Assessment in Adult Neurosurgical Patients Undergoing Posterior Fossa Tumor Resection in the Park Bench Position: A Comparison Between Pulse Pressure Variation (PPV) and Central Venous Pressure (CVP) Guidance |
| --- | --- |
| Principal investicator’s name | List of Authors  1. Pathomporn Pin-on (the first and corresponding author)  Email: [pinon.pathomporn@gmail.com](mailto:pinon.pathomporn@gmail.com)  Tel: +66 86 8970 00 9  2. Srisuluk Kacha Email: [Srisuluk.ka@cmu.ac.th](mailto:Srisuluk.ka@cmu.ac.th) Tel: +66 84 619 6942  3. Ananchanok Sarinkarinkul Email: [asaringc@yahoo.com](mailto:asaringc@yahoo.com) Tel: +66 94 629 6109  4. Nakan Thanakitithum Email: [Nakarn.tana@cmu.ac.th](mailto:Nakarn.tana@cmu.ac.th) Tel: +66 83 958 5680  Affiliation: Department of Anesthesiology  Faculty of Medicine, Chiang Mai University  Chiang Mai, Thailand  The address is 110 Intrawaroros Road, Sriphum, Mueng, Chiang Mai 50200, Thailand |
| Date of submission | September 13, 2024 |
| version | 2 |

**Details of the research proposal outline**

- **Title of the study**

Fluid Responsiveness Assessment in Adult Neurosurgical Patients Undergoing Posterior Fossa Tumor Resection in the Park Bench Position: A Comparison Between Pulse Pressure Variation (PPV) and Central Venous Pressure (CVP) Guidance

- **Principal investigator**

Associate Professor Dr. Pathomporn Pin-on
Affiliation: Department of Anesthesiology, Faculty of Medicine, Chiang Mai University
Contact address: Department of Anesthesiology, Faculty of Medicine, Chiang Mai University, 110 Intawaroros Road. Intawaroros, Subdistrict. Sri Phum Subdistrict City, Chiang Mai Province Chiang Mai 50200
Email: pinon.pathomporn@gmail.com
Phone: 086-897-0009

- **Subinvestigators**

1. Srisuluk Kacha Email: [Srisuluk.ka@cmu.ac.th](mailto:Srisuluk.ka@cmu.ac.th) Tel: +66 84 619 6942
2. Ananchanok Sarinkarinkul Email: [asaringc@yahoo.com](mailto:asaringc@yahoo.com) Tel: +66 94 629 6109
3. Nakan Thanakitithum Email: [Nakarn.tana@cmu.ac.th](mailto:Nakarn.tana@cmu.ac.th) Tel: +66 83 958 5680

- **Name of sponsor, total budget awarded and period of award**

Research sponsor: None
Research funding source: None

- **Background and rationale**

Fluid management plays an important role in the perioperative care of neurosurgical patients, particularly those undergoing posterior fossa tumor resection [1,2]. In these procedures, patients are often placed in the park bench position to optimize surgical exposure and surgical access, which significantly impacts hemodynamics and presents challenges for maintaining adequate tissue perfusion [3,4]. Effective fluid management is essential to prevent complications such as cerebral hypoperfusion or edema, which can have serious complications for patient outcomes [1].

Multiple methods have been employed to guide fluid administration intraoperatively. Pulse Pressure Variation (PPV) and Central Venous Pressure (CVP) monitoring emerge as commonly used approaches. PPV, derived from arterial pressure waveform analysis, reflects the dynamic changes in stroke volume induced by mechanical ventilation, offering a real-time assessment of fluid responsiveness and has been proposed as a reliable indicator of fluid responsiveness in various surgical settings [5,6,7]. In contrast, CVP monitoring has traditionally been utilized to guide fluid therapy. However, its reliability is limited due to variations in intrathoracic pressures. Because preload depends on ventricular volumes, CVP serves as an inadequate predictor, with its ability to accurately predict fluid responsiveness being approximately 56% [8,9,10].

Posterior fossa tumor resection often requires the park bench position, characterized by the patient being placed with their head rotated and tilted downward [11,12]. This positioning alters venous return and cardiac output. Venous return may be compromised and potentially leading to decreased preload. This can result in decreased stroke volume, cardiac output and blood pressure [3]. Inadequate fluid administration may lead to hypovolemia, impaired tissue perfusion, and hemodynamic instability, while excessive fluid administration can result in cerebral edema, increased intracranial pressure, and other cardiovascular complications. To prevent this, careful monitoring and adjustment of fluid status and precise fluid management strategies may be necessary.

This study aims to address this knowledge gap by investigating PPV and CVP guidance in fluid management during posterior fossa tumor resection, in the park bench position. By assessing the hemodynamic response to fluid challenges and to correlate them with clinical outcomes, we aim to investigate the difference in fluid responsiveness assessment between PPV and CVP in this patient population.

- **The objectives of the trial or study, its hypotheses or research questions, its assumptions, and its variables**

Question:

- What are the differences in intraoperative fluid administration volumes between PPV and CVP-guided strategies during posterior fossa tumor resection in the park bench position?

**Objective**

- To quantify the differences in intraoperative fluid administration volumes between Pulse Pressure Variation (PPV) and Central Venous Pressure (CVP) guidance in adult neurosurgical patients undergoing posterior fossa tumor resection in the park bench position
  - Incidence of hypotension
  - Compare intraoperative hemodynamic parameters, including mean arterial pressure (MAP) and heart rate (HR), between the PPV-guided and CVP-guided fluid management strategies.
  - Assess the amount of intraoperative fluid administered in the PPV-guided group compared to the CVP-guided group.
  - Determine the incidence of intraoperative complications, such as hypotension or fluid overload, associated with each fluid management strategy

**Hypothesis**

- There is a significant difference in fluid responsiveness assessment between Pulse Pressure Variation (PPV) and Central Venous Pressure (CVP) guidance during posterior fossa tumor resection in adult neurosurgical patients in the park bench position.
- **Study type**

This study is a prospective single-center, single-blind randomized trial conducted at Maharaj Nakorn Chiang Mai Hospital.

- **Study outcomes**
  1. Primary outcome: Total intraoperative fluid administration volumes during surgery
  2. Secondary outcomes

Intraoperative period

- - - Hemodynamic profiles: Mean arterial pressure (MAP), SBP, DBP
    - Incidence of hypotension intraoperatively, measured from systolic blood pressure (SBP) that is fall from baseline 20% at any time during surgery or MAP < 65 mmHg
    - Amount of fluid administration
    - Blood transfusion
    - Serum lactate level
    - Vasoactive drug requirement

Postoperative period

- - - Glasglow coma score at 24 hours after surgery
    - Vasoactive drug requirement
    - Total ventilator days
    - Length of neurosurgical intensive care unit stay
- **Study population**
  - Source of participants:
    - Adult patients undergoing posterior fossa tumor resection in the park bench position at Maharaj Nakorn Chiang Mai Hospital between September 24, 2024 – September 25, 2025 will be prospectively evaluated
  - Number of participants and sample size calculation – Sample size is calculated based on previous study of Janani Gopal et al [13]. The average fluid volume administration of patients in the CVP and PPV group were 4,300 and 3,500 ml, respectively. Alpha is 0.05, Beta is 0.2, and power of 80%. The sample size is 25 patients/ group.


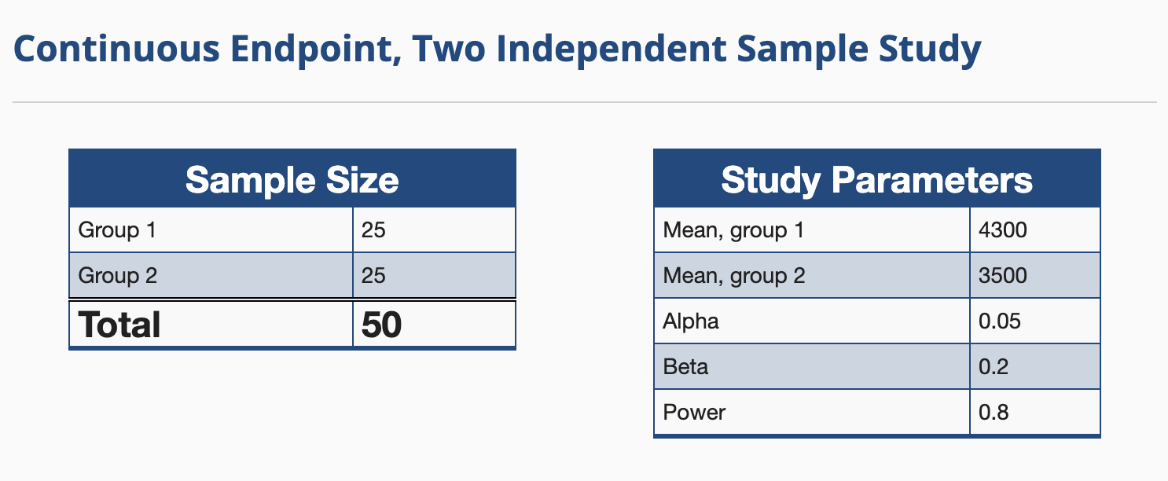


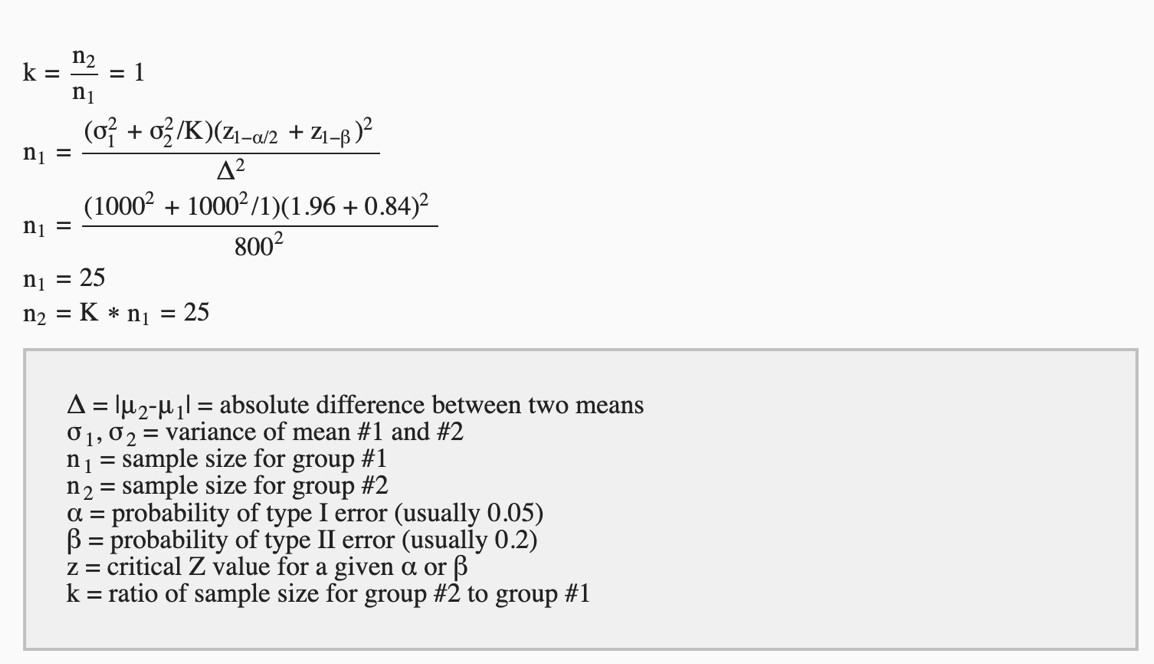


*https://clincalc.com/stats/samplesize.aspx*

- - Eligibility criteria
    - Inclusion criteria
      1. Adult patients aged 18 years and above
      2. American Society of Anesthesiologists (ASA) I and II
      3. Diagnosis of posterior fossa tumor requiring surgical resection in the park bench position, under general anesthesia at our hospital
      4. Willing to participate in the study
    - Exclusion criteria
      1. Arrhythmia
      2. Significant cardiac diseases
      3. Chronic obstructive airway disease
      4. Elevated intra-abdominal pressure
      5. Tumors prone to precipitate diabetes insipidus
      6. Peripheral vascular disease
      7. Pulmonary hypertension
      8. Patients in sepsis
- Withdrawal criteria
- None
  - The expected duration of subject participation: one week of the admission in the neurosurgical intensive care unit after posterior fossa tumor resection.
- **วิธีการวิจัย (Methods)**
  1. The research tools used in this study - Data recording forms
  2. Methods
  - **Randomization:** Randomization of participants was carried out by an independent statistician with no involvement in the study. Using computer-generated random numbers, 54 patients were divided into PPV group or CVP group in blocks of four. Allocation ratio is 1:1. Patients in the CVP group were administered fluids guided by CVP measurements, whereas those in the PPV group received fluids guided by PPV measurements. Allocation concealment was ensured through sequentially numbered opaque envelopes, which were opened at the time of admission to the operating room.
  - **Blinding:** Participants were blinded to group assignment throughout the study duration, while anesthesiologists and researchers were not blinded. Data collection was conducted by attending anesthesiologist who was aware of the group allocation, but not involved in further study. The statistical analysis was done by a statistician who was not part of the study.
  - **Anesthetic management**

After patients were transferred to the operating room, standard ASA monitoring was promptly initiated, including electrocardiography, pulse oximetry (SpO2), and noninvasive blood pressure (BP) monitoring via cuff. Additionally, bispectral index (BIS) electrodes were applied for depth of anesthesia monitoring. To ensure adequate venous access, a peripheral line of 18 gauge or larger was promptly established.

Anesthesia was induced with fentanyl 1 to 2 mcg/kg, and propofol 1.5 to 2 mg/kg. Tracheal intubation was facilitated with cisatracurium 0.15-0.2 mg/kg, followed by maintenance via an infusion of cisatracurium at a rate of 1-2 mcg/kg/min. Anesthesia was maintained with propofol-based total intravenous anesthesia administered via Target-Controlled Infusion (TCI), with a targeted effect-site propofol concentration of 2.5–4.0 μg/ml titrated to maintain a Bispectral Index (BIS) of 40 to 60. Analgesia was managed with intermittent boluses of 1 mcg/kg fentanyl. Mechanical ventilation was administered with a minimum tidal volume of 8 ml/kg and a respiratory rate set at 10 to 16 breaths/min to maintain an end-tidal carbon dioxide level between 30 and 35 mmHg.

After induction, an arterial catheter was placed in the radial artery for invasive BP monitoring. A triple-lumen central venous catheter was inserted in the internal jugular vein using ultrasound guidance for CVP monitoring. Both CVP and PPV were measured by Mindray monitor. Baseline values of CVP, PPV, and serum lactate were recorded in both groups. Baseline PPV measurement was conducted once mechanical ventilation was standardized, with a tidal volume set at 8 ml/kg for all patients.

Normal PPV usually less than 13%. Simultaneously a baseline CVP was also measured. All patients exhibited normal baseline CVP levels ranging from 8-12 cm H2O. Depending on randomization, CVP or PPV monitoring was used intraoperatively to guide fluid management. The corresponding monitor not in use was deactivated until the end of surgery, with the anaesthetist restricted from accessing its data. Mannitol was administered prior to dural opening at a dosage of 0.5-1 g/kg.

Intraoperative parameters including heart rate, SBP, DBP, MAP, CVP, or PPV were documented. Prior to extubation, all patients received ondansetron injection at a dose of 0.1-0.15 mg/kg to prevent postoperative nausea and vomiting. Neuromuscular blockade reversal was achieved using neostigmine (0.05 mg/kg) and glycopyrrolate (0.01 mg/kg). Extubation occurred once patients regained full consciousness. Arterial blood gas analysis was repeated at the end of surgery, with vital signs carefully noted. Subsequently, patients were transferred to the intensive care unit for postoperative monitoring. Follow-up assessments were conducted within the first 24 hours postoperatively to monitor patient recovery progress. Postoperative fluid management was determined by neurosurgeons who were unaware of the patient's group allocation.

- - **Fluid management protocol**

CVP and PPV will be measured and recorded in both groups. Both parameters appear on the monitor screen throughout the operation. As a maintenance fluid, each patient will receive a weight-based formula of 0.9% normal saline solution and lactated Ringer solution [21]. Fluid management protocol will be activated, based on their assigned group (CVP or PPV), as shown in Fig 2. The treatment goal for the PPV group is a PPV value of less than 13%, whereas the treatment goal for the CVP group is a CVP value between 8-12 mmHg [22, 23]. Before the implementation of the fluid management protocol, acute blood loss exceeding the permissible threshold and/ or hemoglobin < 9 gm/dl must be addressed and treated with pack red cell (PRC) transfusion [24]. The maximum allowable blood loss (MABL) is determined by considering gender, weight-based estimated blood volume, preoperative hematocrit, and the minimum acceptable hematocrit for the patient.


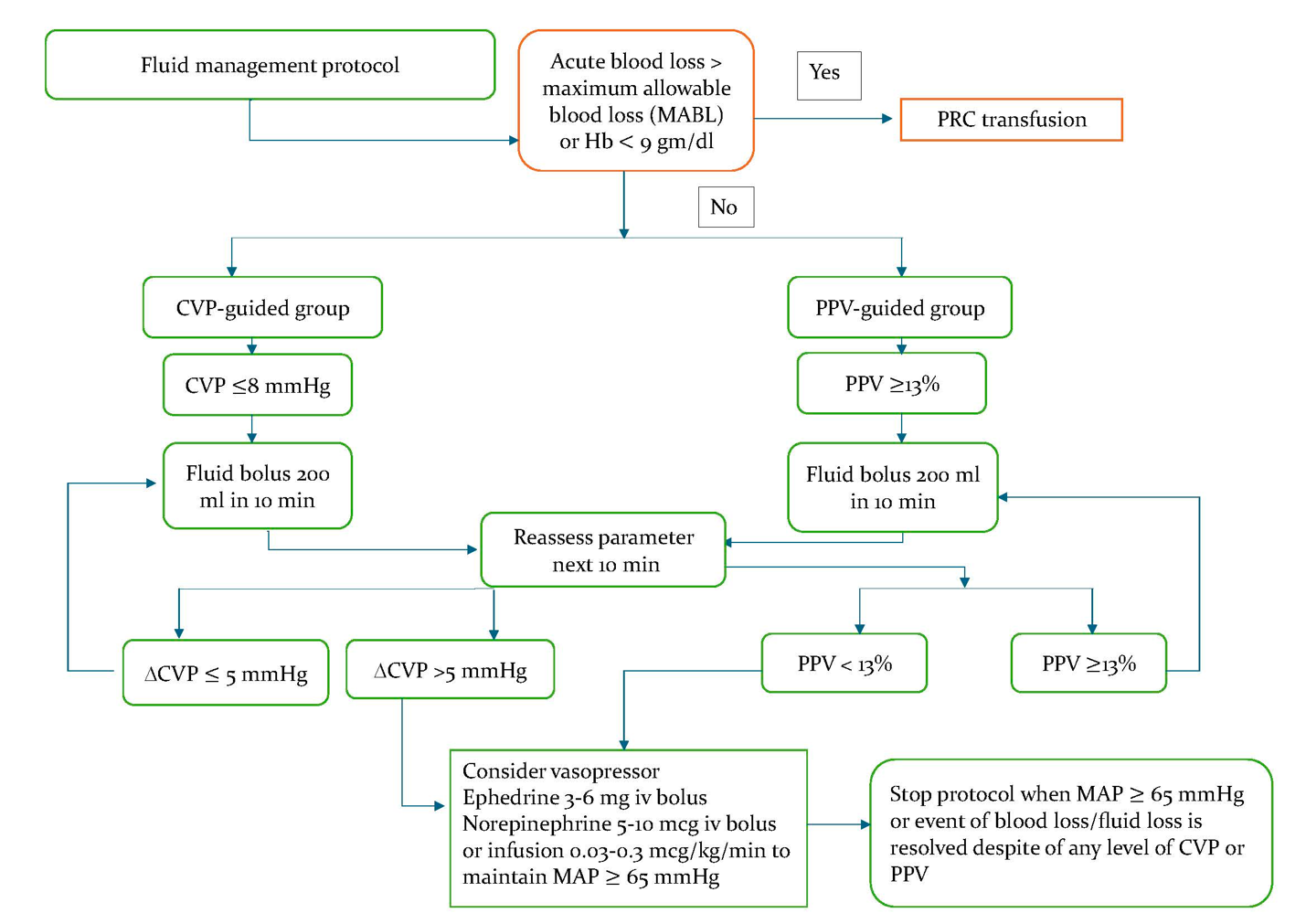


3. Retrieve information from medical records in the computer system. The target population for the study is adult patients undergoing posterior fossa tumor resection in the park bench position at Maharaj Nakorn Chiang Mai Hospital December 1, 2024 – May 31, 2025.

3.1 Data is gathered from pre-anesthetic record, intraoperative anesthetic record, and neurosurgical intensive care unit record. All document is scanned into the electronic medical record (EMR) system of Maharaj Nakorn Chiang Mai Hospital.

3.2 Section 1 General information and preoperative variables, including gender, age, weight, height, size of tumor, systolic blood pressure, diastolic blood pressure, heart rate, Glasgow Coma Scale score, ASA classification, and laboratory (Hb, Hct, platelet count, Na, K, Cl, HCO_3_, BUN, Cr, Albumin)

3.3 Section 2 Intraoperative variables, Anesthetic related parameters: including systolic BP, diastolic BP, MAP (measured every 15 minutes), PPV, CVP, heart rate, anesthetic time, anesthetic technique (TIVA or Volatile anesthesia), type and amount of intravenous fluid (crystalloid, colloid, blood product), body temperature, urine output, blood lactate and pH, vasoactive drug administration,Surgical related parameters: including operation time, estimate blood loss, surgeon request of mannitol infusion

3.4 Section 3 Postoperative variables include the length of neurosurgical intensive care unit stay, blood transfusion, vasoactive drug requirement, and total ventilator days

- **Study design**

Prospective single-center, single-blind randomized trial

The variables of interest

| Pre-operation | Intra-operation | Post-operation |
| --- | --- | --- |
| 1. Gender 2. Age 3. BMI 4. ASA classification 5. Glasgow coma score 6. Size of tumor 7. Systolic BP 8. Diastolic BP 9. Heart rate 10. Laboratory (Hb, Hct, platelet count, Na, K, Cl, HCO3, BUN, Cr, Albumin) | 1. SBP 2. DBP 3. MAP 4. Heart rate 5. PPV 6. CVP 7. Operation time 8. Anesthetic time 9. Anesthetic technique: TIVA or Volatile anesthesia 10. Mannitol infusion 11. Inotropic drug/vasopressor 12. Estimate blood loss 13. Type and amount of intravenous fluid (crystalloid, colloid, blood product) 14. Body temperature 15. Urine output 16. Blood lactate 17. pH | 1. The length of neurosurgical intensive care unit stay  2. Blood transfusion  3. Vasoactive drug requirement  4. Duration of ventilator-dependent |

- **Specialized terminology definition**
  - Anesthetic time: The duration during which the patient undergoes general anesthesia until the completion of surgery
  - Operation time : The length of time from the start of a surgical procedure to its completion.
  - The length of neurosurgical intensive care unit stays: The duration of time a patient remains in the specialized ICU following neurosurgical procedures.
  - Duration of ventilator-dependent: The length of time a patient relies on mechanical ventilation to support their breathing. This duration begins from the time when the patient is first placed on a ventilator and ends when they are successfully weaned off and no longer require mechanical assistance to breathe adequately.
- **Plans for data collection and statistical analysis; stopping rule or premature termination of research, if applicable**
  - The researchers presented the research proposal and data collection form to the research committee for approval
  - The researchers coordinated with the anesthetic team at Maharaj Nakorn Chiang Mai Hospital to explain about the research progress
  - The researchers collected and analyzed data, collecting data from anesthetic records and medical records

|  | Aug  2024 | Sep  2024 | Oct  2024 | Nov  2024 | Dec 2024 to May 2025 | | | | | Jun  2025 | Jul  2025 | Aug  2025 | Sep  2025 | Dec  2025 |
| --- | --- | --- | --- | --- | --- | --- | --- | --- | --- | --- | --- | --- | --- | --- |
| Ethic approval |  |  |  |  |  |  |  |  |  |  |  |  |  |  |
| Data collection |  |  |  |  |  |  |  |  |  |  |  |  |  |  |
| Statistical analysis |  |  |  |  |  |  |  |  |  |  |  |  |  |  |
| Manuscript drafting |  |  |  |  |  |  |  |  |  |  |  |  |  |  |

- **Data analysis and statistical methods**

**Descriptive statistics**

Statistical analysis involves continuous data presented as mean, median, and standard deviation using the student t-test, depending on data distribution. Categorical data will be displayed as counts and percentages, analyzed using the Chi-square test or Fisher’s exact test

**Analytic statistics**

The continuous outcome variable will be compared using independent two-sample T-test, if they are normally distributed. If they are non-normally distributed, the medians from two groups will be compared using Mann-Whitney U and Wilcoxon rank sum test. The statistical significance is indicated by P values < 0.05. Data analysis will be conducted using IBM SPSS version 22.0 (Armonk, NY: IBM Corp) program.

- **Ethical issues and considerations**
  - **Justification for the study, its significance**

To investigate the difference in fluid responsiveness assessment between PPV and CVP during posterior fossa tumor resection in adult neurosurgical patients in the park bench position to be utilized in improving patient care.

- - **Regulatory and Ethical Compliance**

This research study will adhere to international ethical principles and guidelines, following the principles of the Declaration of Helsinki (2013) and Good Clinical Practice (GCP) guidelines, including the "Guidelines for Human Research" of the Faculty of Medicine, Chiang Mai University. The research project will undergo review by the Research Ethics Committee of the Faculty of Medicine, and will commence after receiving approval from the research ethics committee. Any amendments to the research protocol will be submitted for approval before proceeding.

- - **Informed consent and participant recruitment**

This prospective single-blind randomized trial was conducted at Maharaj Nakorn Chiang Mai Hospital from December 1, 2024, to May 31, 2025. Prior to enrollment, patients were provided with detailed information about the study protocol, and written informed consent was obtained preoperatively during the pre-anesthetic evaluation in the patient’s ward by the researcher. Participants were informed of their right to withdraw from the study at any time without needing to provide a reason. All research procedures adhered to the ethical standards outlined in the Helsinki Declaration.

- - **Risks of participants as a result of participation in a research study**

This study is prospective single-blind randomized trial, which increases clinical risk to patients. This research project falls under research that poses moderate risk, as the data collected will be stored securely in the computer of the research unit, accessible only with a password. The risk assessment typically includes the following factors:

- Surgical and Anesthetic Risks: All participants in this study will undergo posterior fossa tumor resection in the park bench position, which inherently carries surgical and anesthetic risks, including the possibility of vascular injury, nerve injury, brain edema, respiratory complication and hemodynamic instability. However, the procedures involved in this research do not introduce additional risks beyond those normally associated with such surgeries.
- Specific Research-Related Risks: The primary research intervention involves the continuous monitoring of hemodynamic using CVP and PPV. Complications of central venous catheter and arterial catheter can occur during the insertion or use of the catheter, including pneumothorax, cardiac arrhythmias, arterial injury, infectious complication and thrombotic complication. Preventing complications involves strict adherence to aseptic techniques during catheter insertion, proper site selection, use of ultrasound guidance to avoid mechanical complications, and regular monitoring of the catheter site. Infections can be minimized with the use of sterile dressing changes.

The researcher will adhere to the "Guidelines for the Use of Medical Record Data for Research" of the Faculty of Medicine, Chiang Mai University, and will implement measures to maintain confidentiality as follows:

- The data recording form has been designed to include the least amount of detail necessary to achieve the research project's objectives.
- The data collected will be stored securely in the computer of the research unit, accessible only with a password.
- The researcher will retain and maintain the data for a period of 10 years, after which the files will be securely deleted from the computer used for storage.
- If there is a need to send the dataset to external units or individuals outside the Faculty of Medicine, Chiang Mai University, the researcher must prepare a Data Use and Sharing Agreement between the receiving party and the Faculty of Medicine. The dataset sent to the recipient must be in a format that does not identify individuals or contain personal information (Limited Data Set).
  - **Anticipated benefits to research participants or society**

Although this study may not directly benefit the participants, it has provided valuable insights into significant difference in fluid responsiveness assessment between Pulse Pressure Variation (PPV) and Central Venous Pressure (CVP) guidance during posterior fossa tumor resection in adult neurosurgical patients in the park bench position and to correlate them with clinical outcomes. These findings serve as fundamental data for monitoring and preventing various complications. This, in turn, can lead to the enhancement of patient care processes in the future.

- **Confidentiality**

The personal information of the research participants will be kept confidential and anonymized, using codes instead of the participants' names and surnames. Only the researchers will have access to the data of the research participants.

- - **Planning after the recruitment finish**

After the research is concluded, there are plans to publish the research findings in medical journals. When publishing the research results in academic journals, data sharing may occur according to the journal's requirements in a format that cannot be linked to personally identifiable information. This is aimed to benefit the development of patient care. Additionally, there are plans for further research to build upon the findings of this study in the future.

- **References**
  1. Ryu T. (2021). Fluid management in patients undergoing neurosurgery. Anesthesia and pain medicine, 16(3), 215–224. <https://doi.org/10.17085/apm.21072>
  2. Jeker, S., Beck, M. J., & Erb, T. O. (2022). Special Anaesthetic Considerations for Brain Tumour Surgery in Children. Children (Basel, Switzerland), 9(10), 1539. <https://doi.org/10.3390/children9101539>
  3. Rozet, I., & Vavilala, M. S. (2007). Risks and benefits of patient positioning during neurosurgical care. Anesthesiology clinics, 25(3), 631–x. <https://doi.org/10.1016/j.anclin.2007.05.009>
  4. Mavarez-Martinez, A., Israelyan, L. A., Soghomonyan, S., Fiorda-Diaz, J., Sandhu, G., Shimansky, V. N., Ammirati, M., Palettas, M., Lubnin, A. Y., & Bergese, S. D. (2020). The Effects of Patient Positioning on the Outcome During Posterior Cranial Fossa and Pineal Region Surgery. Frontiers in surgery, 7, 9. <https://doi.org/10.3389/fsurg.2020.00009>
  5. Rathore, A., Singh, S., Lamsal, R., Taank, P., & Paul, D. (2017). Validity of Pulse Pressure Variation (PPV) Compared with Stroke Volume Variation (SVV) in Predicting Fluid Responsiveness. Turkish journal of anaesthesiology and reanimation, 45(4), 210–217. <https://doi.org/10.5152/TJAR.2017.04568>
  6. Biais, M., Ehrmann, S., Mari, A., Conte, B., Mahjoub, Y., Desebbe, O., Pottecher, J., Lakhal, K., Benzekri-Lefevre, D., Molinari, N., Boulain, T., Lefrant, J. Y., Muller, L., & AzuRea Group (2014). Clinical relevance of pulse pressure variations for predicting fluid responsiveness in mechanically ventilated intensive care unit patients: the grey zone approach. Critical care (London, England), 18(6), 587. <https://doi.org/10.1186/s13054-014-0587-9>
  7. Teboul, J. L., Monnet, X., Chemla, D., & Michard, F. (2019). Arterial Pulse Pressure Variation with Mechanical Ventilation. *American journal of respiratory and critical care medicine*, *199*(1), 22–31. <https://doi.org/10.1164/rccm.201801-0088CI>
  8. Marik, P. E., Baram, M., & Vahid, B. (2008). Does central venous pressure predict fluid responsiveness? A systematic review of the literature and the tale of seven mares. *Chest*, *134*(1), 172–178. <https://doi.org/10.1378/chest.07-2331>
  9. Marik, P. E., & Cavallazzi, R. (2013). Does the central venous pressure predict fluid responsiveness? An updated meta-analysis and a plea for some common sense. *Critical care medicine*, *41*(7), 1774–1781. <https://doi.org/10.1097/CCM.0b013e31828a25fd>
  10. Sundaram, S. C., Salins, S. R., Kumar, A. N., & Korula, G. (2016). Intra-Operative Fluid Management in Adult Neurosurgical Patients Undergoing Intracranial Tumour Surgery: Randomised Control Trial Comparing Pulse Pressure Variance (PPV) and Central Venous Pressure (CVP). Journal of clinical and diagnostic research: JCDR, 10(5), UC01–UC5. <https://doi.org/10.7860/JCDR/2016/18377.7850>
  11. Bălașa, A.F., Hurghiș, C., Tămaș, F., & Chinezu, R. (2020). Patient Positioning in Neurosurgery, Principles and Complications. Acta Marisiensis - Seria Medica, 66, 14 - 9.
  12. Sathyanarayanan J., Hari K. (2014), Anaesthetic considerations for posterior fossa surgery, Continuing Education in Anaesthesia Critical Care & Pain, 14, 202–206, <https://doi.org/10.1093/bjaceaccp/mkt056>
  13. Gopal J, Srivastava S, Singh N, Haldar R, Verma R, Gupta D, Mishra P. Pulse Pressure Variance (PPV)-Guided Fluid Management in Adult Patients Undergoing Supratentorial Tumor Surgeries: A Randomized Controlled Trial. Asian J Neurosurg. 2023 Sep 22;18(3):508-515. doi: 10.1055/s-0043-1771364. PMID: 38152505; PMCID: PMC10749863.
