# Supplementary material for: Study protocol for a randomized controlled trial comparing pulse pressure variation (PPV) and central venous pressure (CVP) guidance for fluid responsiveness assessment in neurosurgical patients undergoing posterior fossa tumor resection in park bench position
Source: PLoS One. 2025 Jun 2;20(6):e0324590. doi: 10.1371/journal.pone.0324590 (PMC12129314; doi:10.1371/journal.pone.0324590)
Supplement: S3 File — (DOCX) [file pone.0324590.s003.docx]

**Human Participants Research Checklist**

***Complete the following if your study involved human participants or human participants’ data. These questions should be addressed for prospective and retrospective studies.***

1. Did you obtain ethics approval for this study?
   - If yes, please upload (file type “Other”) the original approval document you received from your ethics committee. If the original document is in another language, please also provide an English translation.

_x__ Uploaded _ N/A

- - If you did not obtain ethical approval, please explain why this was not required below.

Yes, the study protocol has been approved by the Institutional Ethical Committee of Faculty of Medicine, Chiang Mai University. The Ethical Approval document will be uploaded along with the study protocol, the version that has been approved. Together with the informed consent form will be uploaded.

1. If you prospectively recruited human participants for the study – for example, you conducted a clinical trial, distributed questionnaires, or obtained tissues, data or samples for the purposes of this study, please report in the Methods:
   1. the day, month and year of the **start and end** of the recruitment period for this study.
   2. whether participants provided informed consent, and if so, what type was obtained (for instance, written or verbal, and if verbal, how it was documented and witnessed). If your study included minors, state whether you obtained consent from parents or guardians. If the need for consent was waived by the ethics committee, please include this information.

___ Completed __ N/A

The patient recruitment has been planned to recruited since September 2024, however, because of the delayed in the fluid management protocol revision, we plan to recruit the participants in December 1, 2024 till May 31, 2025. If we cannot achieve the number of participants as proposed in the sample size calculation, we will request the institutional ethical review board to extend the period of approval for another 6 months, which counting from September 2025 forward.

1. If you are reporting a retrospective study of medical records or archived samples, please report in the Methods section:
2. the day, month and year when the data were accessed for research purposes
3. whether authors had access to information that could identify individual participants during or after data collection

__ Completed __ X _ N/A
