# Supplementary material for: Study protocol for a randomized controlled trial comparing pulse pressure variation (PPV) and central venous pressure (CVP) guidance for fluid responsiveness assessment in neurosurgical patients undergoing posterior fossa tumor resection in park bench position
Source: PLoS One. 2025 Jun 2;20(6):e0324590. doi: 10.1371/journal.pone.0324590 (PMC12129314; doi:10.1371/journal.pone.0324590)
Supplement: Attachment — Submitted filename: Response to editor.docx [file pone.0324590.s004.docx]

To editorial team of journal PLOS ONE

The following is an explanation of the amendments made to the Study protocol for comparing pulse pressure variation and central venous pressure guidance for fluid responsiveness assessment in neurosurgical patients undergoing posterior fossa tumor resection in park bench position (PONE-D-24-50639) that has been submitted to the journal.

| Suggestions for amendments and additions | Explanation | Notes |
| --- | --- | --- |
| - Please complete all items on the Human Participants Research Checklist - Upload the completed Human Participants Research Checklist as file type “Other” | The Human Participants Research Checklist has been filled and uploaded in the file type of “Other” | Done and re-submit on November 7, 2024 |
| - Please note that the Study Protocol article type is only suitable for proposals of studies that have not yet started or that are ongoing. - Please ensure that your Methods section reports the status and timeline of the study. | - This study protocol is ongoing process, we planned to recruit the participants in September 2024, however, there was a minor revision in the fluid management protocol, therefore, we plan to recruit the patients in January 2025 to May 2026, 18 months. - In the method section, I have added the sentences indicated A) participant recruitment will be completed, B) data collection will be completed and C) results are expected. | Done and re-submit on November 7, 2024 |
| - Please note that you must upload a completed SPIRIT schedule as Fig 1 of your manuscript. | - The SPIRIT schedule of participant enrolment, intervention, and assessment has been uploaded in Fig 1. | Done and re-submit on November 7, 2024 |
| - Please upload a copy of your study protocol that was approved by your ethics committee/IRB as a Supporting Information file. - The study protocol, including the complete and detailed plan for the conduct and analysis of the trial that was approved by the ethics committee/IRB. | - The ethical approval document from the Institutional Ethical Committee of the Faculty of Medicine, Chiang Mai University has been uploaded together with the study proposal (version 2), which is the version that was approved and also the informed consent form that has been translated from Thai to English language. | Done and re-submit on November 7, 2024 |
| - Our author guidelines for Clinical Trial Study Protocols require that you upload a SPIRIT checklist as Supporting Information. | - The SPIRIT checklist for Clinical Trial Study Protocol will be submitted as one of the Supporting Information | Done and re-submit on November 8, 2024 |
| - “During our evaluation of the documents provided, we noted that your ethics approval letter did not cover the entire date range for participant recruitment and data collection. - Before we can proceed further with the submission, please provide the original ethics document for the study. - If the document is in another language, please also provide an English translation. | - We have explained the proposed timeline of participant recruitment and data collection to be relevant to the period of approval by the institutional ethical committee in the “Research site and anticipated schedule”, page 4, line 98-107. - Here is the detail:   **Research site and anticipated schedule**  This research is a prospective, single-center, double-blind randomized trial performed at a tertiary care university hospital in Northern Thailand. The institutional ethics committee approved the study protocol from September 26, 2024, until September 25, 2025.The research team intends to prospectively gather patient data from December 1, 2024, to May 31, 2025, in the brain and spinal cord operating room at Maharaj Nakorn Chiang Mai Hospital, as well as postoperative data from the neurosurgical intensive care unit. Data gathering will conclude by June 2025, with outcome analysis expected to be finalized by December 2025. Should the research team determine, within 45 days prior to the expiration of the ethical committee approval date, that recruitment of the planned patient cohort may be unfeasible, we will submit a request for a 6-month extension of the research approval. | Done and re-submit on November 13, 2024 |

This study protocol is submitted for consideration for publication in PLOS ONE, demonstrating its thorough planning and potential to contribute valuable evidence for anesthesia care in neurosurgery.

Pathomporn Pin-on (Corresponding author)
